# Essential Role for Ethanolamine Plasmalogen Hydrolysis in Bacterial Lipopolysaccharide Priming of Macrophages for Enhanced Arachidonic Acid Release

**DOI:** 10.3389/fimmu.2017.01251

**Published:** 2017-09-29

**Authors:** Luis Gil-de-Gómez, Alma M. Astudillo, Patricia Lebrero, María A. Balboa, Jesús Balsinde

**Affiliations:** ^1^Instituto de Biología y Genética Molecular, Consejo Superior de Investigaciones Científicas (CSIC), Universidad de Valladolid, Valladolid, Spain; ^2^Centro de Investigación Biomédica en Red de Diabetes y Enfermedades Metabólicas Asociadas (CIBERDEM), Madrid, Spain

**Keywords:** lipolysaccharide priming, arachidonic acid, phospholipid remodeling, lipid mediators, eicosanoids, monocytes/macrophages, inflammation

## Abstract

Due to their high content in esterified arachidonic acid (AA), macrophages provide large amounts of eicosanoids during innate immune reactions. Bacterial lipopolysaccharide (LPS) is a poor trigger of AA mobilization in macrophages but does have the capacity to prime these cells for greatly increased AA release upon subsequent stimulation. In this work, we have studied molecular mechanisms underlying this phenomenon. By using mass spectrometry-based lipidomic analyses, we show in this work that LPS-primed zymosan-stimulated macrophages exhibit an elevated consumption of a particular phospholipid species, i.e., the ethanolamine plasmalogens, which results from reduced remodeling of phospholipids *via* coenzyme A-independent transacylation reactions. Importantly however, LPS-primed macrophages show no changes in their capacity to directly incorporate AA into phospholipids *via* CoA-dependent acylation reactions. The essential role for ethanolamine plasmalogen hydrolysis in LPS priming is further demonstrated by the use of plasmalogen-deficient cells. These cells, while responding normally to zymosan by releasing quantities of AA similar to those released by cells expressing normal plasmalogen levels under the same conditions, fail to show an LPS-primed response to the same stimulus, thus unambiguously demonstrating a cause–effect relationship between LPS priming and plasmalogen hydrolysis. Collectively, these results suggest a hitherto unrecognized role for ethanolamine plasmalogen hydrolysis and CoA-independent transacylation reactions in modulating the eicosanoid biosynthetic response.

## Introduction

Sepsis and septic shock produced by Gram-negative bacteria constitute a major cause of morbidity and mortality. Lipopolysaccharide (LPS) plays a central role by stimulating TLR4-expressing cells of innate immunity to produce an inflammatory response (1). Macrophages, as innate immune cells, release large quantities of arachidonic acid (AA) when stimulated with agonists. The free AA is subsequently metabolized to form a wide variety of oxygenated metabolites with key regulatory roles in inflammation (2, 3). While a poor stimulus for AA release on its own, bacterial LPS possesses the capacity of priming the cells for an increased release of AA when the cells are exposed to a second inflammatory stimulus (4–6). Controlled formation of eicosanoids is regarded as beneficial because it helps optimize defensive reactions against the invading microorganisms; however, excessive, uncontrolled production of these compounds, i.e., an eicosanoid storm, can be self-destructive and lead to irreversible damage (3).

Availability of free AA is recognized as a rate-limiting step in the generation of eicosanoids by immune cells (7). The levels of free AA are modulated by two opposing reactions; on one hand, phospholipid deacylation, on the other hand, reacylation back into phospholipids. The fatty acid is excised from the *sn*-2 position of glycerophospholipids by phospholipase A_2_ enzymes, of which group IVA cytosolic phospholipase A_2_α (cPLA_2_α) is the critical one, and reincorporated into phospholipids by the concerted action of acyl-CoA synthetases and CoA-dependent acyltransferases (7). In resting cells, reacylation dominates, hence, the amount of free AA available for eicosanoid production is low. In stimulated cells, the phospholipase A_2_-mediated deacylation reaction dominates, resulting in elevated levels of free AA, which become available to cyclooxygenases and lipoxygenases for enhanced eicosanoid synthesis (7).

Arachidonic acid recently esterified to phospholipids is subjected to further remodeling reactions aimed at placing the fatty acid in the appropriate cellular phospholipid localizations, which seems to be important, among other things, for the correct execution of the eicosanoid biosynthetic response during simulation (7–9). These reactions are catalyzed by CoA-independent transacylase (CoA-IT), which transfers AA moieties from diacyl-phospholipid species to ether phospholipids, particularly the ethanolamine plasmalogens (7–9).

Our laboratory has been delineating the molecular mechanisms underlying AA mobilization in phagocytic cells responding to stimuli of the innate immune response (10–16). We use mass spectrometry-based lipidomic approaches to elucidate, at a molecular species level, the sources of AA involved in the processes of release and reacylation (17), and to uncover new stimulus-specific lipid activation markers whose metabolic pathways of synthesis can provide targets for pharmacological intervention. In this work, we have applied similar approaches to study the process of LPS priming of macrophages for enhanced AA release. We demonstrate that LPS priming causes a marked reduction in the entry of AA into a discrete phospholipid species, i.e., the ethanolamine plasmalogens, which appears to be due to diminished activation of CoA-IT-mediated transacylation reactions. The data suggest a hitherto unrecognized role for CoA-IT in LPS priming and, in turn, emphasize the key role of phospholipid fatty acid remodeling reactions to limit the amount of free AA available for eicosanoid synthesis.

## Materials and Methods

### Reagents

RPMI 1640 and Dulbecco’s modified Eagle’s cell culture media was from Molecular Probes-Invitrogen (Carlsbad, CA, USA). Chloroform and methanol (HPLC grade) were from Fisher Scientific (Hampton, NH, USA). Lipid standards were from Avanti Polar Lipids (Alabaster, AL, USA) or Larodan Fine Chemicals (Malmoe, Sweden). The cPLA_2_α inhibitor pyrrophenone was synthesized and provided by Dr. Alfonso Pérez (Department of Organic Chemistry, University of Valladolid). All other reagents were from Sigma-Aldrich.

### Cell Culture and Stimulation Conditions

Resident peritoneal macrophages from Swiss male mice (University of Valladolid Animal House, 10–12 weeks old) were obtained by peritoneal lavage using 5 ml cold PBS, and cultured in RPMI 1640 medium with 10% heat-inactivated serum, 100 U/ml penicillin, and 100 µg/ml streptomycin, as described elsewhere (18, 19). All procedures involving animals were undertaken under the supervision of the Institutional Committee of Animal Care and Usage of the University of Valladolid, and are in accordance with the guidelines established by the Spanish Ministry of Agriculture, Food, and Environment and the European Union.

RAW264.7 cells and the ether phospholipid-deficient RAW.12 cells (generously provided by Dr. R. A. Zoeller, Boston University) (20, 21) were grown in Dulbecco’s modified Eagle’s medium supplemented with 10% (v/v) fetal bovine serum, 100 U/ml penicillin, 100 µg/ml streptomycin, and 2 mM l-glutamine at 37°C in a humidified atmosphere of 5% CO_2_ at 37°C, as previously described (22).

For experiments, the cells were incubated in fresh serum-free medium for 1 h before addition of various concentrations of LPS (priming step) for different times. Afterward, various concentrations of zymosan were added to the cultures (stimulation step) for different times. When pyrrophenone was used, it was added after the priming step for 10 min before the addition of zymosan.

Zymosan was prepared exactly as described (15, 19). *In vitro* assays were conducted to ensure that the zymosan batches used in this study contained no endogenous phospholipase A_2_ activity (23–27). Cellular protein was measured by the Bradford procedure (28), utilizing a commercial kit (BioRad Protein Assay). Protein levels did not significantly change over the course of the periods of LPS priming and subsequent zymosan exposure.

### Analysis of Glycerophospholipids and by Liquid Chromatography/Mass Spectrometry

This was carried out exactly as described elsewhere (10–16), using a high-performance liquid chromatograph equipped with a binary pump Hitachi LaChrom Elite L-2130 and a Hitachi Autosampler L-2200 (Merck), coupled on-line to a Bruker Esquire 6000 ion-trap mass spectrometer (Bruker Daltonics, Bremen, Germany). Phospholipid molecular species were identified by comparison with previously published data (10–16, 22, 29).

### Analysis of Fatty Acids by Gas Chromatography/Mass Spectrometry

This was carried out exactly as described elsewhere (30–32), using an Agilent 7890A gas chromatograph coupled to an Agilent 5975C mass selective detector operated in electron impact mode, equipped with an Agilent 7693 Autosampler and an Agilent DB23 column (60 m length × 0.25 mm internal diameter × 0.15 µm film thickness).

### Measurement of [^2^H]AA Incorporation into Cellular Phospholipids

The cells were either treated or not with LPS for 1 h before exposing them to zymosan in the presence of exogenous [^2^H]AA (1 µM). The amount of [^2^H]AA in the various phospholipid species was determined by liquid chromatography/mass spectrometry as described above.

### Measurement of Phospholipid [^2^H]AA Remodeling

The cells, either treated or not with LPS, were pulse-labeled with [^2^H]AA (1 µM) for 30 min. Cells were washed four times with bovine serum albumin (medium containing 0.5 mg/ml in culture medium) to remove unincorporated fatty acid. Afterward, the cells were placed in serum-free medium and incubated with zymosan for 1 h. The amount of [^2^H]AA in the various phospholipid species was determined by liquid chromatography/mass spectrometry as described above.

### Statistical Analysis

All experiments were carried out at least three times with incubations in duplicate or triplicate, and the data are expressed as mean ± SE. Statistical analysis was carried out by Student’s *t*-test, with *p* < 0.05 taken as statistically significant.

## Results

### Pretreatment of Macrophages with LPS Increases AA Release Stimulated by Zymosan

**Figure 1:**
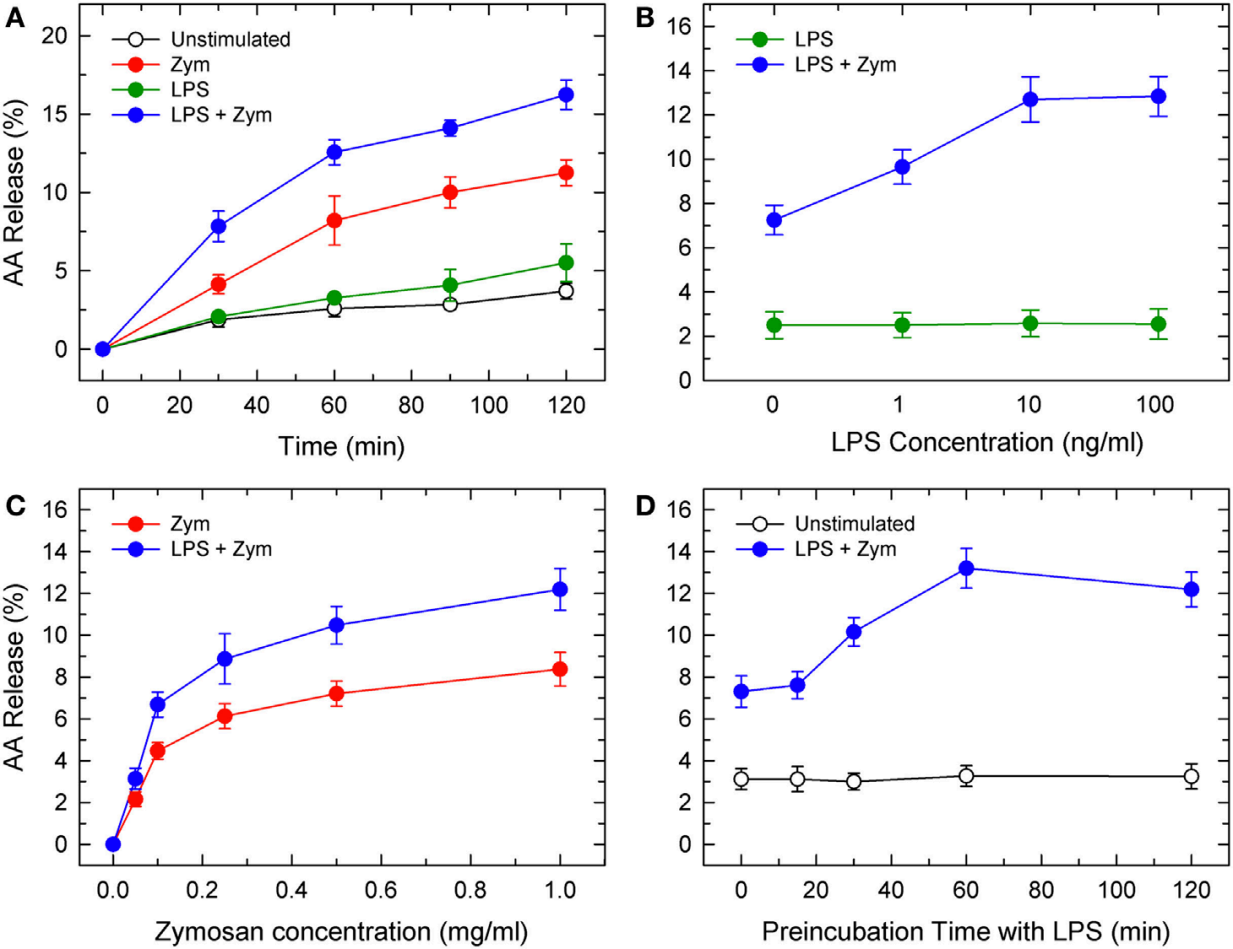
Lipopolysaccharide (LPS) priming of mouse peritoneal macrophages for enhanced AA release in response to zymosan. **(A)** Kinetics of AA release by control (unstimulated, open circles), LPS-primed unstimulated (100 ng/ml LPS for 1 h prior to *t* = 0; green circles), unprimed zymosan-stimulated (1 mg/ml; red circles), and LPS-primed zymosan-stimulated (100 ng/ml LPS for 1 h prior to *t* = 0 followed by 1 mg/ml zymosan for the times indicated; blue circles). **(B)** Effect of LPS concentration. The cells were primed with the indicated concentrations of LPS for 1 h and then stimulated (blue bars) or not (green bars) with 1 mg/ml zymosan for 2 h. **(C)** Effect of zymosan concentration. The cells were either unprimed (red circles) or primed with 100 ng/m LPS (blue circles) for 1 h prior to addition of the indicated amounts of zymosan for 2 h. **(D)** Effect of the duration of preincubation of the cells with LPS. The cells were preincubated with 100 ng/ml LPS for the times indicated. Afterward, the cells were either untreated (open circles) or treated with 1 mg/ml zymosan (blue circles) for 2 h. The amount of AA released is expressed as % mass of fatty acid initially present in the cells. Results are expressed as mean ± SE (*n* = 3).

Previous studies utilizing radioactively labeled AA have demonstrated that pretreatment of phagocytic cells with LPS increases AA mobilization in response to a second stimulus (4–6). Radioactive precursors do not always distribute uniformly among all phospholipid pools; thus, the subsequent AA release response measured may overestimate the contribution of phospholipid sources with higher turnover rates (15). In this study, we have used gas chromatography and liquid chromatography coupled to mass spectrometry to obtain accurate estimates, both qualitative and quantitative, of phospholipid sources of released AA.In response to LPS alone, mouse peritoneal macrophages released no appreciable amounts of AA. However, AA mobilization was remarkably stimulated upon subsequent activation of the macrophages with yeast-derived zymosan (Figure 1A). LPS priming increased by 1.5-fold the zymosan-stimulated AA mobilization response. Preincubation with 1 ng/ml LPS already resulted in enhanced AA release, with a maximum at 10–100 ng/ml (Figure 1B). LPS priming was observed over a broad range of zymosan concentrations (Figure 1C) and required at least a 60-min preincubation period (Figure 1D). LPS priming did not change whether or not serum was present during the incubations.

### Phospholipid Sources for AA Mobilization during LPS Priming

**Figure 2:**
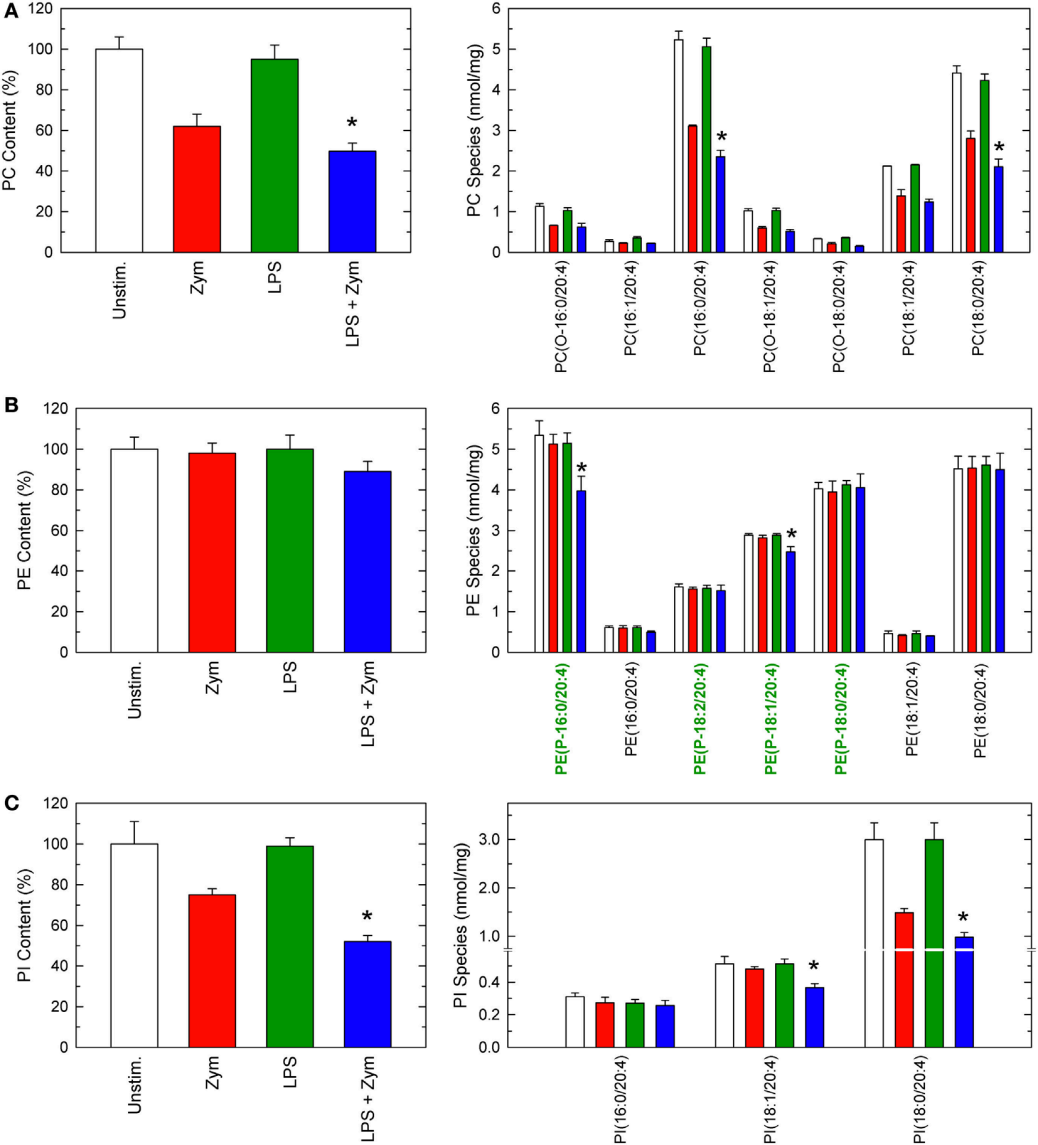
Arachidonic acid (AA)-containing phospholipid species in macrophages. The cells were untreated (open bars and red bars) or treated (green bars and blue bars) with 100 ng/ml lipopolysaccharide (LPS) for 1 h. Afterward, they were not stimulated (open bars and green bars) or were stimulated (red bars and blue bars) with 1 mg/ml zymosan for 2 h, as indicated. The cellular content of AA-containing PC **(A)**, PE **(B)**, or phosphatidylinositol (PI) **(C)** molecular species was determined by liquid chromatography/mass spectrometry. Ethanolamine plasmalogen species in **(B)** are labeled in green in the abscissa for easier identification. Results are expressed as means ± SE (*n* = 4). *Significantly different (*p* < 0.05) from incubations with zymosan but without LPS.

The phospholipid sources for AA mobilization in activated macrophages were determined by liquid chromatography/mass spectrometry (10–16, 22, 29). The profile of AA-containing phospholipids in unstimulated macrophages is shown in Figure 2, and overall agrees with previous results by us (11, 14, 15) and other investigators (33). Ethanolamine plasmalogen species, diacyl PC species, and stearoyl phosphatidylinositol (PI) were the major AA-containing species. Treatment of the cells with LPS did not induce any appreciable change in the content or species distribution of AA (Figure 2). When unprimed cells were stimulated with yeast-derived zymosan particles, marked decreases in the content of cellular AA-containing phospholipids were appreciated (Figure 2). All major AA-containing PC species plus PI (18:0/20:4) contributed to this release. On the opposite side, no PE species significantly changed its AA content.

**Figure 3:**
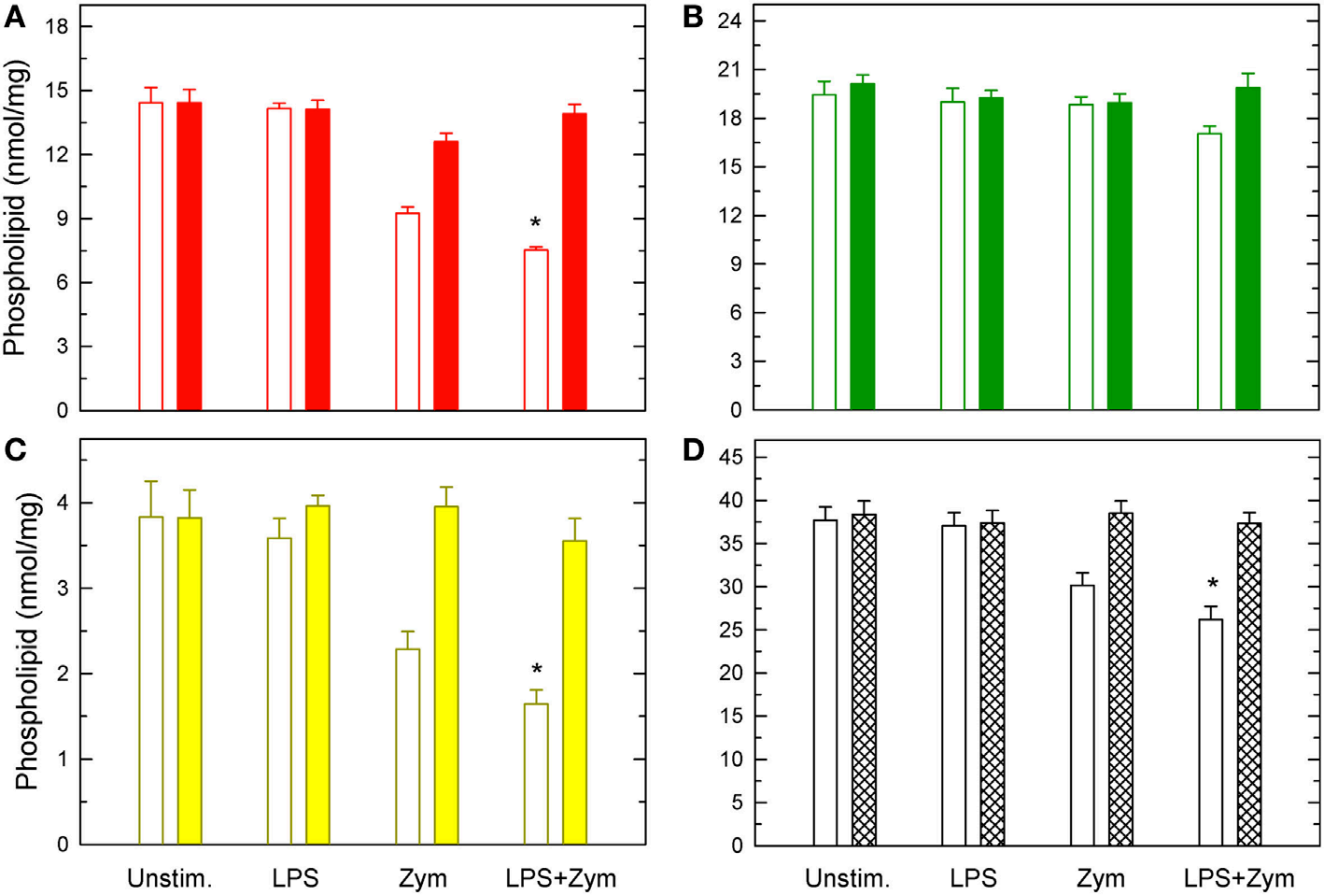
Effect of pyrrophenone on phospholipid deacylation in macrophages. The cells were either untreated or treated with 100 ng/ml lipopolysaccharide (LPS) for 1 h, as indicated. Afterward, they were treated (colored bars) or not (open bars) with 1 µM pyrrophenone for 10 min. Finally, the cells were stimulated with 1 mg/ml zymosan for 2 h, as indicated. Arachidonic acid (AA)-containing PC **(A)**, PE **(B)**, Pl **(C)**, or total AA-containing phospholipids **(D)** was determined by liquid chromatography/mass spectrometry. Results are expressed as means ± SE (*n* = 4). *Significantly different (*p* < 0.05) from incubations with zymosan but without LPS.

Importantly, when the LPS-primed cells zymosan-stimulated cells were analyzed, striking changes were appreciated (Figure 2). Decrease of AA from PC, and PI were more noticeable, but the most conspicuous finding was that, now, decreases were also appreciated in PE, arising primarily, if not solely, from plasmalogen species (Figure 2). Thus, LPS priming facilitates the hydrolysis of plasmalogen PE species after zymosan stimulation. All these decreases were prevented if the zymosan incubations included the well-established cPLA_2_α inhibitor pyrrophenone (Figure 3), thus confirming the general role that cPLA_2_α plays in macrophage AA release.

**Figure 4:**
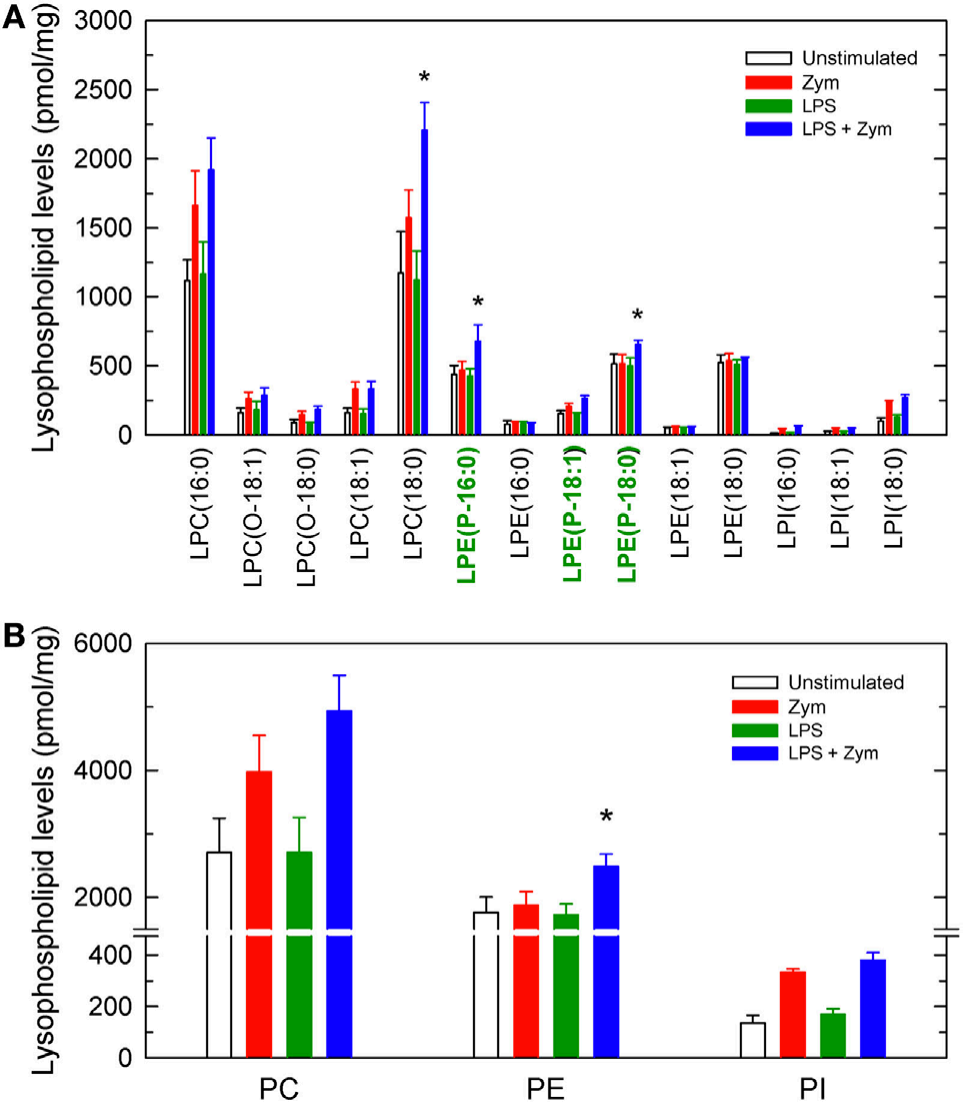
Lysophospholipid molecular species generated by activated macrophages. The cells were untreated (open bars and red bars) or treated (green bars and blue bars) with 100 ng/ml lipopolysaccharide (LPS) for 1 h. Afterward, they were not stimulated (open bars and green bars) or were stimulated (red bars and blue bars) with 1 mg/ml zymosan for 2 h, as indicated. The cellular content of lysophospholipid molecular species **(A)** or total lysophospholipids formed by class **(B)** was determined by liquid chromatography/mass spectrometry. Ethanolamine lysoplasmalogen species in **(A)** are labeled in green in the abscissa for easier identification. Results are expressed as mean ± SE (*n* = 4). *Significantly different (*p* < 0.05) from incubations with zymosan but without LPS.

To complete the picture of changes occurring via phospholipid deacylation reactions, the profile of lysophospholipids formed after zymosan-stimulated cells, both LPS-primed and unprimed, was measured next (Figure 4). Significant increases in a number of lysoPC and lysoPI species were detected, and their levels were further increased in the LPS-primed cells, as expected. Regarding lysoPE species, significant increases of ethanolamine lysophospholipids were observed only in the LPS-primed cells zymosan-stimulated, and involved only the plasmalogen forms (Figure 4).

### Studies with Plasmalogen-Deficient Cells

**Figure 5:**
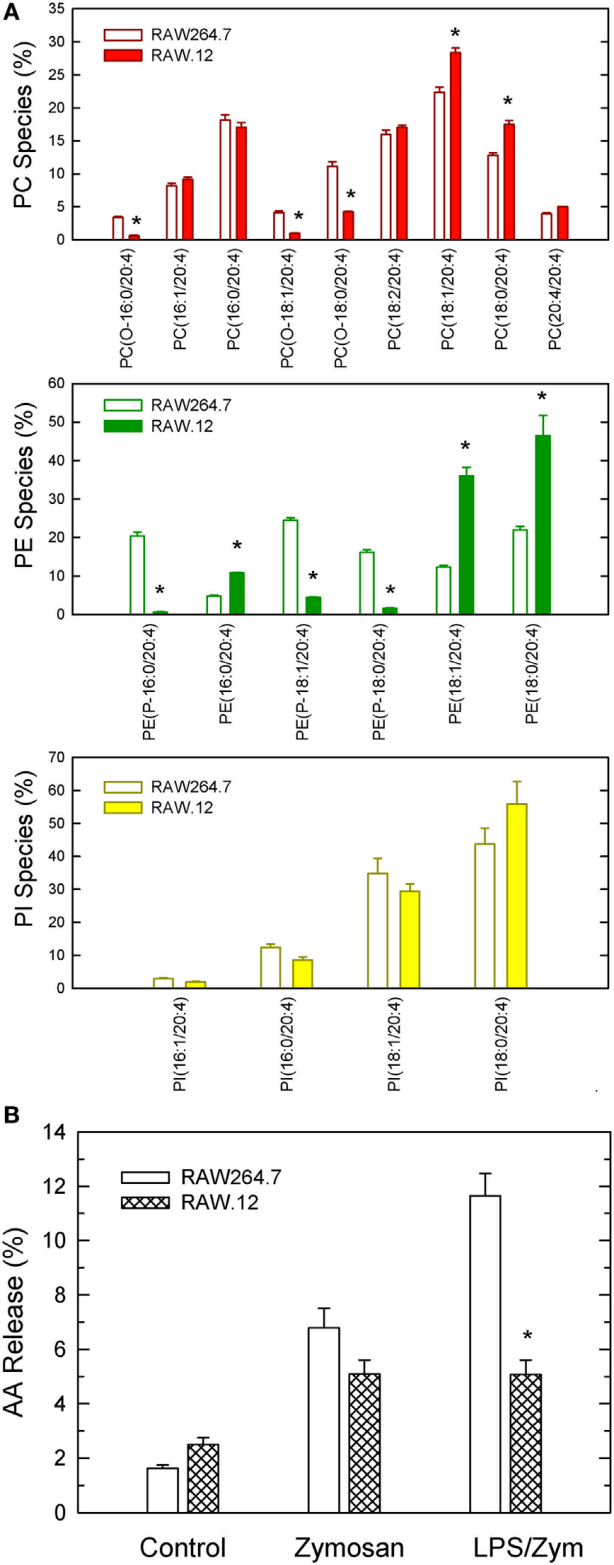
Characterization of plasmalogen-deficient RAW.12 cells. **(A)** Arachidonic acid (AA) distribution profile among PC (red bars), PE (green bars), and phosphatidylinositol (PI) (yellow bars) species of RAW.12 cells. For comparison, the AA distribution among species of RAW264.1 cells is also shown (open bars). **(B)** AA release by RAW 264.7 (open bars) and plasmalogen-deficient RAW.12 cells (stripped bars). The cells were either untreated (control) or treated with 1 ng/ml lipopolysaccharide for 1 h. Afterward, the cells were stimulated with 150 µg/ml zymosan for 2 h, as indicated in the abscissa. Results are expressed as mean ± SE (*n* = 3). *Data in RAW.12 vs data in RAW264.7, significantly different (*p* < 0.05).

Collectively, the above results appear to suggest that plasmalogen hydrolysis is a determinant of LPS priming for enhanced AA release. To obtain further evidence to support this hypothesis, we studied LPS priming in RAW.12 cells, a plasmalogen-deficient cell line that derives from RAW264.7 cells, a murine macrophage-like cell line that is widely used as a paradigm for studies of macrophage lipid signaling and metabolism (34). RAW.12 cells, originally described by Zoeller and co-workers (20, 21), exhibit greatly reduced amounts of plasmalogens due to defects in the enzymes peroxisomal dihydroxyacetone acyltransferase and endoplasmic reticulum Δ1′-desaturase. Despite the considerable absence of ether phospholipids in RAW.12 cells (including ethanolamine pasmalogens and alkyl-PC species), their AA content was found to be the same as that of native RAW264.7 cells, as quantified by gas chromatography/mass spectrometry (13.51 ± 0.52 and 12.74 ± 0.87 nmol/mg protein, respectively; mean ± SE, *n* = 6). Interestingly, the distribution of AA between phospholipid classes was also preserved in the RAW.12 cell compared to native RAW264.7, because of a compensatory elevation of the levels of AA in diacyl species (Figure 5A). Unprimed RAW264.7 released abundant AA when challenged by zymosan (Figure 5B), and the plasmalogen-deficient cell line RAW.12 released only slightly less AA than the parent strain. Importantly however, when the cells were primed with LPS prior to the zymosan stimulation, AA release in RAW264.7 almost doubled, while in the RAW.12 cells it remained the same as with zymosan alone (Figure 5B). These data provide direct evidence that plasmalogen deacylation is a critical step for LPS priming of macrophages for enhanced AA release in response to a second stimulus.

### LPS Priming Does Not Affect AA Reacylation into Phospholipids

**Figure 6:**
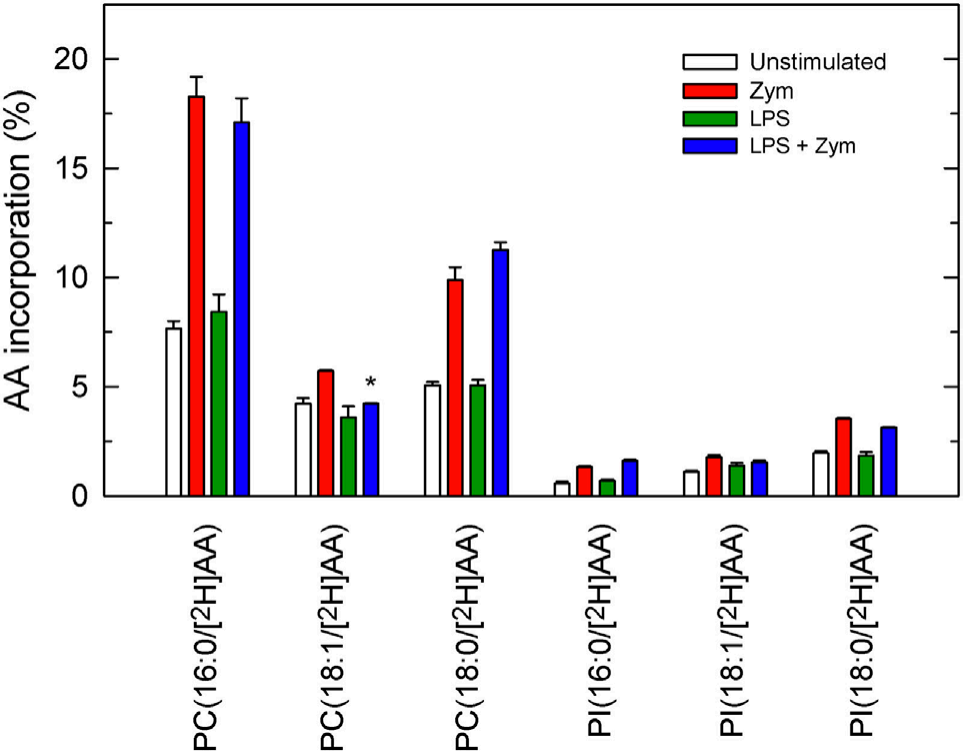
Phospholipid molecular species that incorporate [^2^H]AA. The cells were untreated (open bars and red bars) or treated (green bars and blue bars) with 100 ng/ml lipopolysaccharide (LPS) for 1 h. Afterward, the cells were given 1 µM [^2^H]AA at the time that they were treated without (open bars and green bars) or with 1 mg/ml zymosan (red bars and blue bars) for 2 h. The amount of [^2^H]AA incorporated into the various phospholipid molecular species was analyzed by liquid chromatography coupled to mass spectrometry, and is expressed as % mass of fatty acid initially added to the cells. Results are expressed as mean ± SE (*n* = 3). *Significantly different (*p* < 0.05) from incubations with zymosan but without LPS.

Net AA release in activated cells results from liberation from phospholipids by phospholipase A_2_ minus what is reacylated back by CoA-dependent acyltransferases (7). To evaluate whether LPS priming had any effect on the latter pathway, a liquid chromatography/mass spectrometry metabolipidomic approach using [^2^H]AA to label the initial acceptors involved in CoA-dependent acylation was used (11, 14, 29). This procedure consists in incubating the cells, either LPS-primed or unprimed, with [^2^H]AA at the time they are stimulated with zymosan. Figure 6 shows the species that contained [^2^H]AA after the cells were incubated with zymosan. Most of the [^2^H]AA incorporated into PC species, with minor amounts incorporating into PI species. No PE species incorporated significant amounts of label and, importantly, the profile of [^2^H]AA incorporation into PC and PI was the same, whether the cells were primed with LPS or not.

### Reduced Phospholipid AA Remodeling in LPS-Primed Cells

**Figure 7:**
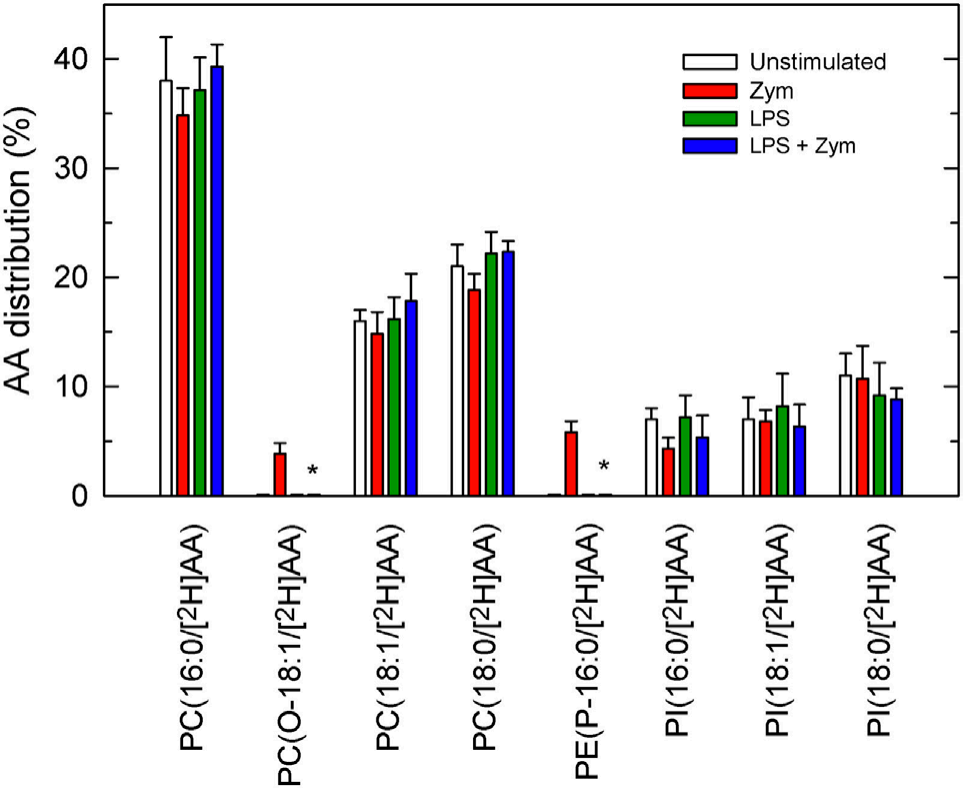
Phospholipid arachidonic acid (AA) remodeling in macrophages. The cells were untreated (open bars and red bars) or treated (green bars and blue bars) with 100 ng/ml lipopolysaccharide (LPS) for 1 h. Afterward, the cells were labeled with 1 µM [^2^H]AA for 30 min. After extensive washing with phosphate-buffered saline to remove the non-esterified [^2^H]AA, the cells were treated without (open bars and green bars) or with (red bars and blue bars) 1 mg/ml zymosan. The distribution of [^2^H]AA among the varies phospholipids species was determined by liquid chromatography coupled to mass spectrometry, and is expressed as % mass of esterified fatty acid. Results are expressed as mean ± SE (*n* = 3). *Significantly different (*p* < 0.05) from incubations with zymosan but without LPS.

In addition to deacylation/reacylation reactions, transacylation reactions between phospholipids are also necessary for AA to distribute properly among membrane phospholipids (7–9). Coenzyme A-independent transacylase (CoA-IT) is the key enzyme and transfers AA between phospholipids, primarily from diacyl-PC species to ethanolamine plasmalogens. To study this pathway, the cells, either LPS-primed or unprimed, were labeled with [^2^H]AA for 30 min. Most of the incorporated fatty acid resided in PC and lesser amounts were found in PI, with no measurable amounts in PE. After the labeling period, the cells were extensively washed to remove the non-esterified [^2^H]AA, and after stimulation with zymosan, the movement of [^2^H]AA from PC to PE species was measured by liquid chromatography/mass spectrometry. As shown in Figure 7, a low but significant amount of label was found in the plasmalogen species PE(P-16:0/[^2^H]AA) after a 1-h incubation with zymosan, confirming the involvement of CoA-IT in the stimulated cells. Similarly, accumulation of label was also found in the ether phospholipid species PC(O-16:0/[^2^H]AA), which is also a preferred acceptor for CoA-IT-dependent transacylation reactions (8). Neither of these species appeared in the LPS-primed zymosan-stimulated cells (Figure 7), strongly indicating that the CoA-IT pathway is blunted by LPS priming.

## Discussion

Although the mechanism of LPS priming for enhanced AA release remains to be fully understood, much effort has been dedicated recently to the characterization of the phospholipase A_2_-mediated deacylation step, leading to a general mechanism for LPS-primed, agonist-stimulated phospholipase A_2_ activation and subsequent AA mobilization that contemplates group IVA cytosolic phospholipase A_2_α (cPLA_2_α) as the key enzyme (35–45). Depending on conditions, a secreted phospholipase A_2_ may also participate by intensifying the cPLA_2_α-mediated response (7, 46). Results from these studies have also shown that LPS priming enhances the basal phosphorylation state of cPLA_2_α in macrophages and neutrophils (6, 47, 48). However, this effect results in little, if any, increases in enzyme activity (5, 6), thus suggesting that the elevation of basal cPLA_2_α phosphorylation is not sufficient to explain the enhanced AA mobilization, and other factor(s) need(s) also be taken into consideration.

Despite all the progress in understanding the phospholipase A_2_-mediated deacylation step, the mechanisms regulating the reacylation and further re-distribution of AA among phospholipids during LPS priming remain obscure. Only recently, the cloning of a number of genes encoding for lysophospholipid acyltransferases (49), has revealed an unanticipated diversity, especially with regard to polar headgroup selectivity. Results from these studies have highlighted a key role for lysophosphatidylcholine acyltransferase 3 as a major controller of AA incorporation in stimulated cells *via* acylation of acyl-PC, thereby limiting free fatty acid available for eicosanoid synthesis (50–52). This view is in agreement with our finding that PC is the major acceptor for AA incorporation in activated macrophages. In our study, no differences in AA incorporation into any phospholipid species were observed between unprimed and LPS-primed cells.

Unexpectedly however, decreased content of AA in some PE species was clearly appreciated in the LPS-primed zymosan-stimulated cells. This is an important finding because PE, despite constituting a major reservoir of AA in unstimulated macrophages, does not behave as a significant acceptor for direct fatty acid reacylation reactions (2, 7–9). Consistent with this view, in our metabolipidomic studies using deuterated AA to analyze direct incorporation of the fatty acid into phospholipids (Figure 6), no significant incorporation into PE species was appreciated.

Arachidonic acid is thought to incorporate into PE molecular species mostly through a series of transacylation reactions regulated by CoA-IT. This enzyme transfers AA primarily from diacyl PC species to ether-linked species, in particular the PE plasmalogens (7–9). This remodeling is thought to be the reason why, when measuring AA mobilization by mass in activated cells, as done in the present study, no changes in the AA content of PE molecular species is observed (10, 14, 15, 33), giving the false impression that PE species do not contribute to zymosan-stimulated AA release. Such contribution is clearly exposed when inhibitors of CoA-IT are used (15). Thus, the rapid and efficient transfer of AA moieties from newly formed AA-containing PC to lysoPE by CoA-IT prevents a decline in the cellular amount of PE during cellular stimulation.

The results of our study using unprimed zymosan-stimulated macrophages are consistent with the involvement of CoA-IT in replenishing PE species with AA as outlined above, since no changes in the mass amounts of PE were detected, while clear decreases were observed in all other phospholipid species. However, in the LPS-primed cells, abundant release of AA from PE, particularly the plasmalogen forms but not the diacyl species, can be readily observed after zymosan stimulation. These findings are confirmed by the elevated levels of lysoplasmenyl-PE species in the LPS-primed cells zymosan-stimulated cells. Thus, PE plasmalogen metabolism emerges as a previously unidentified key event in LPS priming and clearly suggests that diminished recycling of AA into this particular species is responsible for the enhanced AA mobilization response of the LPS-primed zymosan-stimulated cells. Collectively, our results suggest that alterations in CoA-IT-mediated remodeling are central to LPS priming. Compared with unprimed cells, the LPS-primed cells appear to remodel AA from PC to PE more slowly, thus resulting in significantly reduced entry of AA into PE plasmalogens and hence, lower levels of these species. Importantly, these results are relevant to innate immunity and inflammation in that they uncover a hitherto unrecognized LPS-mediated event. Diminished CoA-IT activity after LPS priming may result in excessive damage because of the exacerbated production of eicosanoids subsequent to the increased availability of free AA (3).

Over the last decade, a number of lipid signaling enzymes, including mammalian Mg^2+^-dependent phosphatidate phosphatases (53), platelet-activating factor acetyltransferase (54), and the aforementioned lysophospholipid acyltransferases (49), have been cloned and their sequences determined, which has enabled the application of genetic approaches to unravel their pathophysiological roles. At present, CoA-IT stays as one major enzyme of lipid signaling whose sequence remains elusive. Thus, at present, the only approach to study CoA-IT is the use of activity assays (11, 30). Our initial data in this regard have failed to detect significant decreases of CoA-IT activity in the LPS-primed cells, as measured by a cellular assay (Alma M.Astudillo and Jesús Balsinde, unpublished results). These observations raise the intriguing question of whether LPS priming affects CoA-IT-driven remodeling reactions not by acting directly on the enzyme itself, but by impinging on an unidentified upstream effector that could, for example, decrease the availability of the enzyme to its substrates. This situation would be similar to that described for CoA-IT involvement in platelet-activating factor synthesis by human neutrophils, where the enzyme was suggested to be regulated by substrate availability rather than by increased enzyme activity (55). It is noteworthy that macrophage priming with LPS is strikingly sensitive to inhibitors of transcription and translation, pointing out to the involvement of (a) rapidly turning-over protein(s) (5). Clearly, further studies will be needed to identify factors controlling lysophospholipid and free AA availability during the LPS priming and activation steps.

**Figure 8:**
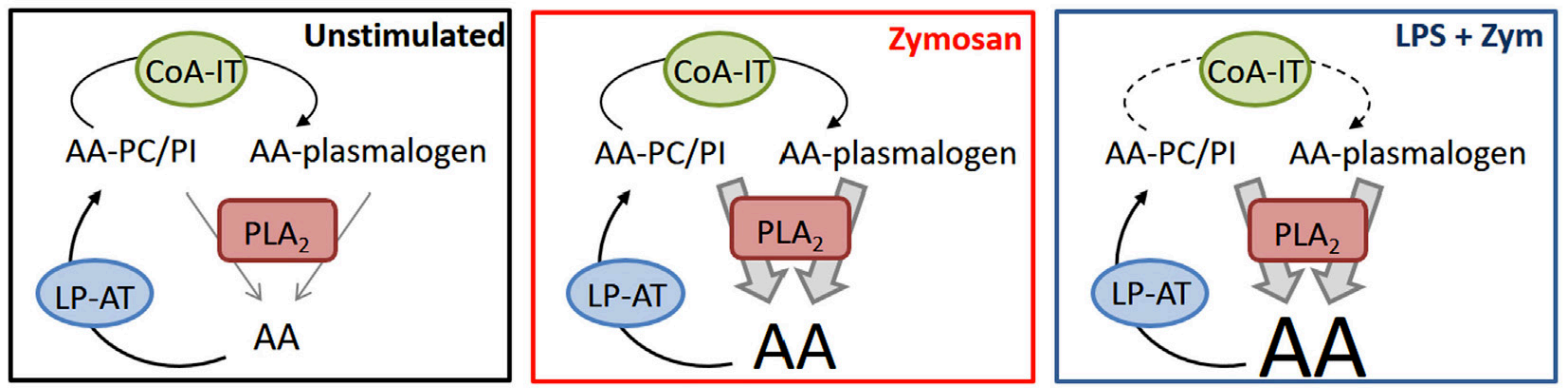
Interactions between CoA-dependent acyltransferases, CoA-independent transacylase, and phospholipase A_2_ in regulating arachidonic acid (AA) release in macrophages. For details, see text. LP-AT, lysophospholipid:acyl-CoA acyltransferase.

In summary, we have uncovered plasmalogen deacylation/reacylation reactions as critical biochemical events of LPS priming of macrophages for enhanced AA release and unveiled a previously unrecognized role for CoA-IT-mediated phospholipid AA remodeling. A schematic representation of our findings is shown in Figure 8. Under resting conditions, little AA is released because esterification reactions mediated by CoA-dependent acyltransferases dominate. Zymosan activates cPLA_2_, which turns the tide, resulting in increased AA release. Such release is further enhanced in LPS-primed cells by a mechanism that slows down the remodeling action of CoA-IT, thus leading to diminished recycling of AA into ethanolamine plasmalogens. Collectively, our results suggest interesting implications in terms of defining previously unidentified targets within the AA remodeling pathway as a strategy to regulate lipid mediator formation.

## Ethics Statement

This study was approved by the Bioethics Committee of the Spanish National Research Council (CSIC) prior to its commencement. All procedures involving animals were undertaken under the supervision of the Institutional Committee of Animal Care and Usage of the University of Valladolid, and are in accordance with the guidelines established by the Spanish Ministry of Agriculture, Food, and Environment and the European Union.

## Author Contributions

LG-d-G, AA, and PL conducted the experiments and interpreted the data. MB designed the experiments and interpreted the data. JB designed the experiments, interpreted the data, and wrote the manuscript. All authors reviewed and approved the manuscript.

## Conflict of Interest Statement

The authors declare that the research was conducted in the absence of any commercial or financial relationships that could be construed as a potential conflict of interest.

## Abbreviations

AA, arachidonic acid; CoA-IT, coenzyme A-independent transacylase; cPLA_2_α, group IVA cytosolic phospholipase A_2_α; LPS, bacterial lipopolysaccharide; PC, choline glycerophospholipid; PE, ethanolamine glycerophospholipid, PI, phosphatidylinositol.
